# Wake Up, Work on Dreams, Back to Bed and Lucid Dream: A Sleep Laboratory Study

**DOI:** 10.3389/fpsyg.2020.01383

**Published:** 2020-06-26

**Authors:** Daniel Erlacher, Tadas Stumbrys

**Affiliations:** ^1^Institute of Sport Science, University of Bern, Bern, Switzerland; ^2^Institute of Psychology, Faculty of Philosophy, Vilnius University, Vilnius, Lithuania

**Keywords:** lucid dream induction, wake-up-back-to-bed, mild, sleep laboratory, morning sleep

## Abstract

Lucid dreaming offers many opportunities to study consciousness processes. However, laboratory research in this area is limited because frequent lucid dreamers are rare. Several studies demonstrated that different methods of induction could increase the number of lucid dreams. In four field studies, a combination of a wake-up-back-to-bed (WBTB) sleep protocol and a mnemonic technique (MILD) showed promising results. To further investigate the effectiveness of this combined approach, we conducted a sleep laboratory experiment with four different conditions. The general experimental procedure was the following: Participants were awakened after 6 h of sleep from a subsequent REM period and kept awake for 30 or 60 min, during which they were asked to practice MILD or a control task (e.g., reading). Then they returned to bed for a morning sleep period. In the first condition eleven sport students, who attended a seminar on sleep and dreams, spent one night in a sleep laboratory. To avoid biases due to the seminar attendance (e.g., higher motivation), in the second condition 15 participants who did not attend the seminar were recruited. In the third condition, 14 sport students were tested with a shorter awakening period (30 min). Finally, the fourth condition served as a control condition, whereas eleven sport students slept two non-consecutive nights in a laboratory. Instead of MILD, in one night the participants read a book (fiction, unrelated to dreams), while in the other night they played a Nintendo Wii video game. In the first three conditions, six (54%), eight (53%), and five participants (36%) reported lucid dreams during the morning sleep period, whereas three, (27%), four (27%), and two participants (14%) produced PSG-verified eye signals. In contrast, in the reading condition, only one (9%) participant reported lucid dreams and no eye movements. No lucid dreams were observed in the Wii condition. The findings of the present study show that by using a combination of WBTB and MILD, lucid dreams can be effectively induced in people who are not selected for their lucid dream abilities.

## Introduction

A lucid dream is a dream in which the dreamer is aware that he or she is dreaming and can often consciously influence dream content (LaBerge, 1985). Sleep laboratory studies show that lucid dreaming usually occurs during REM sleep (LaBerge, 1990), however, in some cases lucid dreams have also been found during NREM sleep (Stumbrys and Erlacher, 2012). Lucid dreams are linked with higher levels of automatic nervous system activity (LaBerge et al., 1986), but also with more pronounced H-reflex suppression (Brylowski et al., 1989). Neurophysiological studies found increased activation during REM lucid dreaming especially in frontal and frontolateral regions but also in temporoparietal regions as well as an functional connectivity between those areas (Voss et al., 2009; Dresler et al., 2012; Baird et al., 2018). This specific dream state offers many opportunities to study consciousness processes (Baird et al., 2019) or psychophysiology in general (LaBerge et al., 2018).

In the general population, studies suggest that about a half of the general population had a lucid dream at least once in their lifetime and about one out of five people are having them at least once a month (Schredl and Erlacher, 2011; Saunders et al., 2016). Though, only 1% of general population experience lucid dreams frequently – several times a week (Schredl and Erlacher, 2011). Lucid dreams can start spontaneously, but most people applied different techniques to learn who to lucid dream (cf. Stumbrys et al., 2014).

In the literature different techniques have been proposed to increase the frequency of lucid dreams. In a systematic review by Stumbrys et al. (2012) in total 35 studies were identified which tested induction techniques empirically. Out of the 35 studies 11 were conducted as sleep laboratory studies whereas the other 24 were done as field experiments – in some cases with low methodological quality. While none of the induction techniques were verified to induce lucid dreams reliably, consistently and with a high success rate, some methods showed to be promising. One of such methods is a combination of Mnemonic Induction of Lucid Dreams (MILD) in combination with special sleep-wake-patterns, e.g., when a person wakes up in early morning hours and after a certain period of time goes back to bed and takes a nap, known as wake-up-back-to-bed (WBTB).

Mnemonic induction of lucid dreams is a cognitive technique based on prospective memory training and applied upon awakening from a dream (Stumbrys and Erlacher, 2014). The technique involves the dreamer rehearsing the dream and visualizing becoming lucid in it while setting an intention to remember to recognize that one in dreaming. LaBerge (1980) established MILD when working on his doctoral dissertation. At the baseline, when he did not apply any induction technique, LaBerge had less than one lucid dream per month. When he developed MILD, it increased his lucid dreams frequency to 18–26 lucid dreams per month and up to four lucid dreams per night. Further evidence for the effectiveness of MILD comes from ten studies (Kueny, 1985; LaBerge, 1988; Levitan, 1989, 1990a,b, 1991a; Edelstein and LaBerge, 1992; Levitan et al., 1992; LaBerge et al., 1994; Levitan and LaBerge, 1994) whereas all of them were conducted by LaBerge’s research group (Stumbrys et al., 2012).

When using MILD after an awakening in early morning hours (i.e., in a combination with WBTB), lucid dreams seem to be much more likely during following naps than the night before (Levitan et al., 1992). Furthermore it was shown that when using with MILD, it is most effective to use WBTB for a period of 30–120 min (LaBerge et al., 1994). The shorter periods of wakefulness, such as taking a nap after 10 min (LaBerge et al., 1994) or immediately after awakening (Levitan, 1991a) are less effective for MILD practice. The same is true for longer periods of wakefulness, such as taking a nap after 4 h (Levitan, 1990a) or 14–17 h after the bed time (Levitan et al., 1992).

While all previous MILD + WBTB studies were conducted only as field experiments, we carried out a sleep laboratory study to investigate the effectiveness of this combined technique. The study included four experiments. In the first experiment, we tested the effectiveness of MILD with 60 min of WBTB with sports students who attended a seminar on sleep and dreams. In the second experiment, to eliminate possible biases due to the seminar attendance, the same procedure was repeated with people who did not attend the seminar. In the third experiment, a shorter time interval of sleep interruption was introduced (30 min). Finally, in the fourth experiment in contrast to dreamwork that has been accomplished during the period of awakening in previous experiments, two alternative activities were tested: a cognitive activity (reading) and a balancing exercise (Wii video game).

## Materials and Methods

### Participants

Table 1 shows the description of the samples for the four conditions of the sleep laboratory study. In the condition 1, 3, and 4, the participants were students from Heidelberg University and took part in a weekly seminar about “Sleep and Sports” at the Institute of Sports and Sports Sciences given by one of the authors (DE). Participants for the experiment therefore were self-selected by their interest in dreams and lucid dream research. No exclusion criteria were made. Participation in the laboratory study was part of the seminar requirement, however, participation was not obligatory because alternative course credits could be received. Most of the participants of the second condition were also voluntary students from Heidelberg University, but who did not attend the seminar. At the time of data collection (2010–2011), ethical review and approval was not required for the study on human participants in accordance with the local legislation and institutional requirements. Participants provided written informed consent before the beginning of the study and the experiment was conducted in accordance with the Declaration of Helsinki [Statistics transferred to Table 1].

**TABLE 1:** Participants characteristics.

	**Study condition**					
	**1 (60 min + MILD)**	**2 (60 min + MILD)**	**3 (30 min + MILD)**	**4 (60 min + Reading/Wii)**	**Test statistic**	***p* =**
N (male/female)	11 (6/5)	15 (9/6)	14 (11/3)	11 (5/6)	χ^2^ (3) = 3.13	0.37
Age	23.73 ± 1.49	23.79 ± 2.82	24.86 ± 2.11	24.91 ± 2.17	*F*(3,47) = 1.11	0.35
DRF^a^ (dreams/week)	2.22 ± 1.49	2.37 ± 2.30	2.59 ± 1.74	1.81 ± 2.17	*F*(3,47) = 0.40	0.75
LDRF^b^ (lucid dreams/month)	0.16 ± 0.30	0.37 ± 0.47	0.54 ± 0.70	0.44 ± 0.78	*F*(3,47) = 0.92	0.35

*^*a*^Dream Recall Frequency, ^*b*^Lucid dream recall frequency.*

### Dream Recall and Lucid Dream Recall Frequency

The participants completed a dream questionnaire (cf. Schredl et al., 2014). In this questionnaire dream recall frequency was measured on a seven-point rating scale ranging from “0 - never” to “6 - almost every morning.” Re-test reliability for this scale is high (*r* = 0.85; Schredl, 2004). Units of mornings per week were calculated by recoding the scale to their class means (*0* = 0, *1* = 0.125, *2* = 0.25, *3* = 0.625, *4* = 1.0, *5* = 3.5, *6* = 6.5). Lucid dream recall frequency was measured on an eight-point rating scale ranging from “0 -never” to “7 - several times a week.” Re-test reliability for this scale is high (*r* = 0.89; Stumbrys et al., 2013a). Units of mornings per months were calculated by recoding the scale to their class means (*0* = 0, *1* = 0.042, *2* = 0.083, *3* = 0.25, *4* = 1.0, *5* = 2.5, *6* = 4.0, *7* = 18). A definition was provided to ensure a clear understanding of lucid dreaming: “In lucid dreams, one has awareness that one is dreaming during the dream. Thus it is possible to wake up deliberately, or to influence the action of the dream actively, or to observe the course of the dream passively” (for the importance of a clear definition, see Snyder and Gackenbach, 1988).

### Polysomnography

In all experiments, polysomnography (PSG) was conducted to register sleep stages. PSG recording included electroencephalogram (EEG: F3, F4, C3, C4, CZ, O2, O1), electroocculogram (EOG), submental electromyogram (EMG), and electrocardiogram (ECG). EEG electrodes were placed according to the international Ten-Twenty system (Jasper, 1958). A XLTEK Trex longtime EEG recorder was used to record sleep data with a DC amplifier and sample rate of 250 Hz. Sleep stages were manually scored according to the AASM criteria (Iber et al., 2007).

### Mnemonic Induction of Lucid Dreams (MILD)

Mnemonic induction of lucid dreams is based on the ability to remember and perform future actions (i.e., prospective memory). It works best after a spontaneous awakening with dream recall. From this dream different events or objects that are highly improbable or bizarre should be identified and could thus be used to recognize the experience as a dream (so-called dream signs). Afterward, while lying in bed and returning to sleep, the individual has to visualize the dream and upon encountering a dream sign imagine oneself becoming lucid and set an intention to remember: “Next time I’m dreaming, I will remember to recognize that I’m dreaming” (LaBerge et al., 1994; Stumbrys and Erlacher, 2014). For the experimental night MILD was introduced to the participants for the first time. The technique was embedded in the wake period of the WBTB procedure and was divided into three parts: (1) writing the dream report; (2) finding dream signs; (3) practicing MILD.

### Procedure

Before the sleep laboratory night, participants received information about the study night and the goals of the study. All steps of the procedure were explained in a written form and participants provided written informed consent.

**FIGURE 1:**
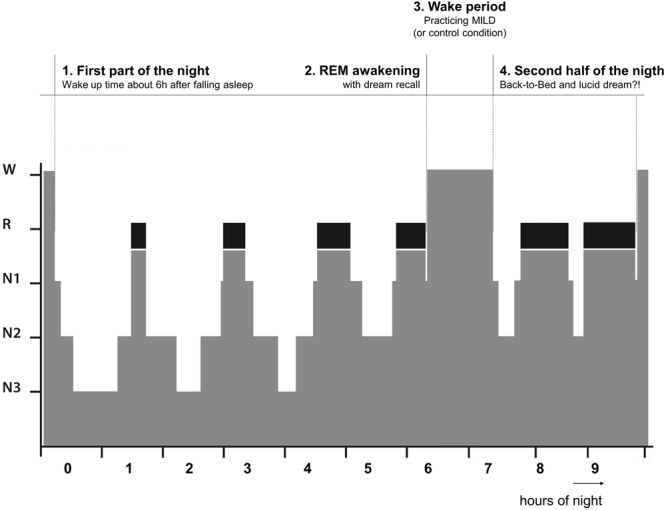
The night procedure divided into two parts.

In conditions 1–3, the participants spent a single night and in condition 4 the participants spent two non-consecutive nights in a dark and quiet room at the Institute of Sports and Sports Sciences (Heidelberg University) with continuous PSG recording. They arrived at 9:00 pm and the experimenter familiarized them with the room and setting. Then the participants prepared themselves for the night and all electrodes were attached by the experimenter. After the recording signals were checked, the experimenter explained to the participants the definition of a lucid dream and trained them in left-right-left-right (LRLR) eye movements to signal a possible lucid dream (cf. LaBerge, 1990). The LRLR signal was trained in front of the recording screen to give the feedback to the participants. The participants were also instructed about the awakening after about 6 h of sleep (see below). The night procedure was divided into four parts (See Figure 1).

#### First Part of the Night

The first part of the night lasted at least 5 h and 40 min after sleep onset. Then the participants were awakened from the subsequent REM period following 10–15 min of uninterrupted REM sleep. If all subsequent uninterrupted REM sleep was shorter than 10 min, the participant was awakened following the next REM period after 7 h from sleep onset, even if it was shorter than 10 min. Further, if a LRLR signal was observed on the sleep recording, the participant was also awakened (3 epochs after the last signal).

#### REM Awakening

Via intercom system, the participants were called by their name until responded. Then they were asked to report any mental content that was in their mind before awakening. If the participant did not recall any sleep mentation immediately, he or she was given 2 min to think about it and try to recall it. Further, the participants were asked if in the dream they were aware that they are dreaming (self-rating of lucidity) and if they gave a LRLR eye-signal. All conversations were recorded via a voice recorded.

#### Wake Period

After awakening the wake period followed. In Experiments 1 and 2, the participants were kept awake for 60 min. During this time period, firstly, the participants were given a dream report sheet and a pen to write down the dream that was just verbally reported (or some vivid earlier dream if nothing was recalled). Then they were given an information sheet about the dream signs (incongruous elements of a dream indicating that this might be a dream, e.g., an odd form, action, context) and asked to go through their dream report and identify all possible dream signs. Lastly, the participants were given a description of MILD technique and asked to practice it with using the present dream report and identified dream signs. To ensure the participants’ clear understanding of dream signs and MILD technique, they were asked to explain both the identified dream signs and MILD technique to the experimenter (and corrected if necessary). The participants in Experiment 3 did exactly the same procedure but with a shorter duration (30 min in total; about 10 min for each step).

The participants in Experiment 4 were also kept awake for 60 min and, in a randomized and counterbalanced order, one night were given a book to read for 60 min (fiction, a collection of short stories, “Hauptsache von Herzen” by Brigitte Sinhuber), while on the other night they played a series of Wii video games that involved body balancing (ski-slalom, snowboarding, etc.) for 60 min. After the wake period finished, the participants returned to bed. The participants in Experiments 1–3 were instructed to keep practicing MILD while falling asleep, whereas the participants in Experiment 4 were simply instructed to recognize that they dreaming the next time they dream.

#### Second Part of the Night (Back-to-Bed)

Upon returning to bed, the participants were further awakened following these conditions: (1) 15 min of uninterrupted REM sleep after 3 h; (2) end of a shorter than 15 min REM period after 4 h; (3) after observing a LRLR eye-signaling on the sleep recording (3 epochs after the last signal). The awakening was made in the same way as before (see above).

All recorded dream reports were transcribed, randomly permutated and scored by a blinded judge for lucidity on a 3-point scale (0 – no evidence of a lucid dream, 1 – possible indications of a lucid dream, 2 – clear indication of a lucid dream), which was shown to have a good interrater agreement (Stumbrys et al., 2013b).

### Criterion for Successful Lucid Dream Induction

A successful induction of a lucid dream could be shown by three types of proofs (see also Schmid and Erlacher, 2020): (1) self-rating of lucidity; (2) an external rater judged the dream report as either with clear or possible indications of lucidity; (3) the participant reported LRLR eye signaling and the eye signals can be unambiguously identified on the sleep recording during REM. For the “strict” criterion, all three criteria must be met. For the “loose” criterion, (1) and (2) were considered as sufficient.

### Statistical Analysis

Because this was an exploratory study, the main focus is on descriptive statistics.

## Results

### Sleep Data

The WBTB sleep data for all conditions is provided in Table 2. Of all 62 experimental nights in the present study, one participant (Experiment 2) was not able to fall asleep after WBTB. The average WBTB sleep latency for all experimental conditions was 31.5 ± 26.0 min. In 53 occasions (85.5%) the participants had REM sleep with an average latency of 42.1 ± 24.7 min after sleep onset. Notably, one participant (Experiment 2) reported a lucid dream after a nap without REM sleep.

**TABLE 2:** Sleep data for the second half of the night.

	**Study condition**					**ANOVA**	
	**1 (60 min + MILD)**	**2 (60 min + MILD)**	**3 (30 min + MILD)**	**4 (60 min + Reading)**	**4 (60 min + Wii)**	***F***	***P***
Total bed time (min)	206.234.5	167.965.4	190.447.5	182.623.4	195.332.3	1.30	0.28
Total sleep time (min)	162.463.5	113.364.1	152.942.3	151.424.5	132.055.2	1.86	0.13
Sleep efficiency (%)	76.622.5	66.022.9	81.013.2	83.110.0	66.423.2	2.21	0.08
Sleep latency (min)	17.010.6	43.931.2	35.635.3	19.39.2	37.017.2	2.76	0.04
REM latency (min)	35.516.7	48.218.9	30.120.1	34.226.2	54.939.5	1.97	0.11
REM period count	2.31.1	1.00.8	2.41.0	2.01.0	1.71.1	4.16	0.01
REM period range	1–4	0–3	1–5	0–4	0–3		
REM total time (min)	36.922.6	29.133.8	47.821.6	35.219.0	28.520.9	1.34	0.27
REM% SPT	20.110.0	20.018.8	31.711.2	22.712.3	17.512.1	2.19	0.08
Wake% SPT	13.715.6	21.926.2	6.28.3	8.39.7	18.418.8	1.93	0.12
Stage 1% SPT	14.67.5	17.112.4	10.27.6	9.06.3	10.76.1	2.04	0.10
Stage 2% SPT	44.713.1	35.919.6	44.311.6	49.28.5	43.514.1	1.53	0.21
Stage 3% SPT	2.83.9	2.54.5	4.75.7	8.19.3	6.86.2	1.95	0.12

### Dream Reports

In total, 115 dream reports were collected during the experimental night: 60 from the first part of the night and 55 from the second part of the night. The dream recall rate for the first part of the night was 95% (from 63 REM awakenings) and for the second part of the night was 76% (from 63 morning naps). The dream reports had an average length of 120.3 ± 121.3 words.

### Induction of Lucid Dreams

In total, the participants reported lucid dreams during 20 morning naps following awakening (32.3%). Further, on four occasions (6.5%) they were unsure if they were dreaming or not. On 14 occasions (22.6%) no dreams were recalled and on 24 occasions only non-lucid dreams were reported (38.7%). The judge rated 24 dream reports as without evidence of lucid dreaming (exactly the same ones as the dreamers themselves), 22 dream reports as with clear indications of lucid dreaming (19 of which the participants rated as lucid and 3 as ambiguously lucid) and two dream reports as with possible indications of lucid dreaming (one which was rated by a participant as lucid and one as ambiguously lucid).

Further, on 14 occasions (22.6%) the participants reported that they produced a LRLR eye signal to confirm their lucidity. In nine cases LRLR eye signals were clearly observed on the PSG recording to occur during unequivocal REM sleep; in three cases the signal and/or sleep stage was ambiguous and in two cases there were no signs of prearranged eye-signaling on the sleep recording. On five occasions (8.1%), the participants reported that they are unsure if they produced a LRLR eye signal. In two of those cases there were unequivocal signals during REM sleep observed on the sleep recording, one case was ambiguous and in two other cases no prearranged eye-signaling was observed. On further five occasions (8.1%), the participants reported that they did not give the signal despite the fact that they were aware of dreaming during the dream. The numbers of lucid dreams according to both “strict” and “loose” criteria in different conditions are presented in Table 3.

**TABLE 3:** Number of lucid dreams in different conditions.

	**Study condition**				
	**1 (60 min + MILD)**	**2 (60 min + MILD)**	**3 (30 min + MILD)**	**4^a^ (60 min + Reading)**	**4^a^ (60 min + Wii)**
N^b^ (male/female)	11 (6/5)	15 (9/6)	14 (11/3)	11 (5/6)	
LD (loose)^c^ (male/female)	6 (2/4)	8 (4/4)	5 (5/0)	1 (1/0)	0
LD (strict)^c^ (male/female)	3 (2/1)	4 (1/3)	2 (2/0)	0	0

*^*a*^Control condition. ^*b*^Number of participants included in the condition. ^*c*^Three types of proofs were used to establish successful induction: (1) self-rating of lucidity, (2) assessment of the dream report by an external judge (3) LRLR eye signals on the sleep recording during REM. For the “strict” criterion, all (1)–(3) had to be met, while for the “loose” criterion only (1) and (2).*

### Condition 1 – 60 Minutes Plus MILD

Six out of 11 participants (54.5%) reported to have a lucid dream in the nap following awakening. All these dreams were verified as lucid by an external judge who scored dream reports. Four participants reported that they produced a LRLR signal (three signals were successfully verified on the PSG recording to occur during unambiguous REM sleep; one signal was ambiguous). Two other participants were unsure if they produced a signal (one signal, however, was verified on the PSG; other signal was ambiguous).

### Condition 2 – 60 Minutes Plus MILD

Eight out of 15 participants (53.3%) reported a lucid dream during the nap. All these dreams were verified as lucid by an external judge who scored dream reports. Six participants reported that they produced a LRLR signal and four of these signals were successfully verified on the PSG recording. In one case, the signal on the PSG recording was ambiguous, in the other case the signal was absent and there were no REM sleep during the nap period.

### Condition 3 – 30 Minutes Plus MILD

Five out of 14 participants (35.7%) reported a lucid dream during the nap and two of them gave a LRLR signal (verified on the sleep recording). Two others did not give a signal and one was awakened on making a signal. One participant reported to make a signal but was uncertain if he was dreaming and corresponding PSG recording showed high EEG alpha levels.

### Control Conditions

In the 60 min plus reading condition, only one participant reported a lucid dream, but did not make a LRLR signal. One other participant was uncertain if he was dreaming and made a signal, however, the signal was verified on the PSG recording.

In the 60 min plus Wii condition, two participants were unsure if they had a lucid dream. One of them reported a dream in a dream and told that he made a signal, the other participant was unsure about signaling. No signals were visible on the PSG recording in both cases.

Taken together conditions 1–4, no gender differences were found for successfully induced lucid dreams with respect neither to the loose (Chi2 = 0.80; *p* = 0.37) nor strict criterion (Chi2 = 0.46; *p* = 0.50). Furthermore, successful participants in having a lucid dream (loose criterion) tended to have a higher baseline dream recall frequency and lucid dream recall frequency compared to the unsuccessful participants, however, this tendency was not statistically significant (*p* = 0.15 and *p* = 0.10, respectively).

## Discussion

The findings of the present study show that by using a combination of WBTB and MILD techniques, lucid dreams can be effectively induced in people who are not selected for their lucid dream abilities. According to the present results, the most effective approach is to use 1 h WBTB time, during which dreamwork is carried out and MILD is practiced. Under such circumstances, about a half of the participants report a lucid dream and about one out of three participants have a lucid dream which could be objectively verified by volitional eye signaling on the sleep recording. Shorter WBTB durations might be less beneficial, as well as if different activities than dreamwork are used during the WBTB period.

The achieved success rates are quite high, if compared to other sleep laboratory lucid dream induction studies with unselected student samples. For example, in a study by Paul et al. (2014), the success rates for visual and tactile stimulation were only 0–7.4%. Our success rates resemble the ones from WBTB + MILD field studies with lucid dreamers by LaBerge, Levitan and their colleagues (Levitan, 1990b, 1991a,b; Levitan et al., 1992; LaBerge et al., 1994). While sleep laboratory and field studies can not be directly comparable (for example, in the former, a researcher can awaken the participant from REM sleep to increase the chances for successful dream recall), this suggests that WBTB + MILD can effectively applied not only by frequent lucid dreamers but also by infrequent or non-lucid dreamers. In the first our experiment, out of four participants who never had a lucid dream before, two became lucid in a single night at the sleep laboratory (two out of seven in the second experiment, but four others did not recall any dream content).

The duration of WBTB period seems to be an important factor in the effectiveness of technique. Previous research showed that with MILD, the most efficient periods of WBTB are of 30–120 min (Levitan, 1990a; Levitan et al., 1992; LaBerge et al., 1994). The findings of the present study indicate that WBTB for 1 h might be more efficient than a shorter period of 30 min. The similar finding was reported by LaBerge et al. (1994), which suggests that 1 h of wakefulness might be the most optimal time for this technique.

Two recent sleep laboratory studies applying an acoustic cue during the induction technique of the WBTB-paradigm might shed some light on the timing issue. In the first study lucid dreams were successfully induced in a single nap session by cueing beeping tones with cognitive training (Carr et al., 2020). The session duration was 20 min and performed in the morning either at 7:30 am or 11:00 am. The results showed that 50% of the cued participants produced a signal-verified lucid dream. In the second study a combination of music (e.g., “Boléro” by Maurice Ravel) with reality testing was applied in 1 h session which was embedded in a WBTB-protocol at 4.5 h after sleep onset (Schmid and Erlacher, 2020). In contrast, only 14% of the participants became lucid and none of those lucid dreams were verified by LRLR eye signal. Thus, it seems that not only the duration of the session but also the hours of previous sleep might be important to enhance the chances to experience a lucid dream.

In contrast to the suggestion by LaBerge (1980) that “it is not the particular activity (carried out during the period of wakefulness), but the alert wakefulness that facilitates lucid dreaming during subsequent sleep” (p. 1042), the present findings indicate that the activity does matter. In our fourth study, where two alternative activities for dreamwork were used (reading and a balancing task), the success rates were markedly lower. A previous study by Leslie and Ogilvie (1996) showed that increased vestibular activation can facilitate dream lucidity, however, in the present study we found no difference between the balancing task and the reading condition. In comparison to reading, the balancing exercise had more disturbing effects on subsequent sleep (increased sleep latency and reduced sleep efficiency). While American Academy of Sleep Medicine (AASM, 2014) lists a vigorous exercise close to bedtime as one of the factors that can increase arousal and disturb sleep, empirical findings are inconsistent (e.g., Stutz et al., 2018). From the present findings, dreamwork (writing down the dream, identifying dream signs, practicing MILD) can be recommended as the optimal activity during the WBTB period.

The period of wakefulness in early morning hours did not disturb subsequent sleep: In only one case (1.6%) the participant was not able to fall asleep after WBTB and in most cases (85.5%) the participants had REM sleep. Interestingly, one participant reported a lucid dream after a nap without REM sleep. While there were no eye-signaling in this case, this might have been an NREM lucid dream, which were also infrequently observed before (Stumbrys and Erlacher, 2012). The participants in the Experiment 2 had longer sleep latency than the participants in the same condition in the Experiment 1. This might be explained by the fact that the Experiment 2 participants in contrast to other groups, did not attend the seminar and therefore might have had higher anxiety/stress level (e.g., due to unfamiliar environment, procedures) which might have resulted in poorer their sleep quality. Yet, the participants in the Experiment 2 achieved very similar lucidity success rates as the ones in the Experiment 1, which suggests that the effectiveness of the present induction method was not influenced by the participation in the seminar (e.g., interest in dreams and/or lucid dreams) and the findings might be more generalizable.

Some methodological issues have to be acknowledged. One of the main challenges in all lucid dream induction studies is what to consider a valid criterion for successful induction (see Stumbrys et al., 2012 for further discussion on this point). In the present study, we employed different measures: the dreamer’s self-report if he/she was lucid and made a LRLR eye movements and the external ratings for dream lucidity based on the dream report and unambiguous LRLR eye signaling during REM sleep. While in the most cases the self-ratings and the external ratings corresponded, on a few occasions they diverged. On three occasions the judge rated dream as clearly lucid whereas the dreamer was unsure if the dream was lucid or not and on one occasion the judge rated a dream as uncertainly lucid whereas the dreamer considered the dream as lucid. Regarding dream lucidity, in such cases we followed the self-report of the dreamer, as the dream lucidity might not be easily inferred from a dream report if it is not explicitly mentioned (e.g., “I became lucid” or “I realized this is a dream”). Yet, if the dreamer was unsure if he was lucid in a dream or awake or if he/she made a LRLR eye signal, but the signal was unambiguously present during REM sleep, we also considered this as a lucid dream. Our previous research (Stumbrys et al., 2014) showed that lucid dreamers quite often are not able to recall their previous waking intentions in lucid dreams and successfully execute them (most often due to hindrances with the dream environment or a premature awakening). While unambiguous eye-signaling on the sleep recording and confirmatory dream report can be considered as the most valid evidence for the confirmation of lucid dreaming, it might not be appropriate to disqualify completely those dreams in which a person was lucid but, for example, forgot to signal or was awakened during the signaling. The conventional minimal criterion for the definition of lucid dreaming is only awareness of dreaming during dreaming (see Stumbrys et al., 2012), while eye-signaling involves also elements of waking memory retrieval and dream body control. Therefore we think it is useful to introduce two aforementioned types of criteria: loose – for expert-validated self-reported experience, and strict – for its objective external validation.

Some further limitations should be acknowledged. Even though 51 participants were included in the study, the sample sizes across the groups are rather small. Indeed, this is one of the reasons, why the results are of descriptive nature. However, the number of about 50% of participants who successful induced a lucid dream within a single sleep laboratory night provides a good reference to what might be a good induction rate in future studies. Furthermore, it should be mentioned that that only one independent judge rated the dream reports, but this was in high accordance with the self-ratings of the participants. Finally, no adaptation night have been done. Therefore, the so-called first night effect might have possible effects on the REM-NREM sleep cycles, e.g., reducing or delaying REM sleep (Agnew et al., 1966).

To summarize, the present study showed that by using a combination of WBTB and MILD, lucid dreams can be effectively induced in people who are not selected for their lucid dream abilities. Future studies should focus on the time of practicing MILD and on combining WBTB with other cognitive techniques (like reality testing) to check their influence on lucid dream induction.

## Data Availability Statement

The datasets generated for this study are available on request to the corresponding author.

## Ethics Statement

Ethical review and approval was not required for the study on human participants in accordance with the local legislation and institutional requirements. The patients/participants provided their written informed consent to participate in this study.

## Author Contributions

Both authors listed have made a substantial, direct and intellectual contribution to the work, and approved it for publication.

## Conflict of Interest

The authors declare that the research was conducted in the absence of any commercial or financial relationships that could be construed as a potential conflict of interest.
